# The Management of Children in the Dental Chair

**Published:** 1885-09

**Authors:** A. T. Bigelow

**Affiliations:** St. Paul


### The Management of Children in the Dental Chair.

Read before the Minnesota State Dental Association, August 1, 1885.

BY A. T. BIGELOW, D.D.S., ST. PAUL.

The real need of constant and skillful care of children's teeth, from the early age of three years, is out of all proportion to its seeming importance. This granted, the query naturally arises as to the best manner of getting control of our little patients.

These fresh specimens from the human fold come palpitating with dire expectations and blanched with fear, led by the blind but inexorable parent like a lamb to the slaughter. The young imagination, always vivid, is frequently heightened by mischievous persons who take delight in seeing the big eyes, as the terrors of the dental chair are narrated.

A toothache usually occasions the first call and the sufferer enters with painfully tense nerves and a sad heart. If the dentist loves children, the required service will be more easily rendered. The child's confidence should be first secured and religiously kept—a searching glance at the operator detects with unerring instinct whether there be kindness—if found, the victory is half won.

A tender and sympathetic manner, without brusqueness, sooths the perturbed spirit and facilitates the examination.

If extraction be required a local anæsthetic may be employed, quietly giving the information that some pain will be felt. This frankness is better for the present and the future. It is cruel and immoral to deceive a child. Cases occur where the most ingenious efforts fail to overcome innate timidity and apprehensiveness. The greatest difficulty is found in filling operations. It is almost a physical impossibility for some children to remain quiet for five consecutive minutes, and dental work cannot be done in an instant. The inability to keep still is constitutional with many, an inheritance peculiarly American.

The adroit use of keen, well tempered instruments, with frequent brief rests, proves least tasking. Boys of a mechanical turn of mind must see every instrument, a permitted look at the one in hand with the assurance that a more curious one will be used at the end of three minutes, secures a short working interval.

Some young boys will submit heroically to the necessary manipulations if allowed to hold a bit of ribbon attached to the dental engine. The idea of driving imparts a sense of stoutness which seems to be a diverting and encouraging principle in such instances.

The elevator often proves a convenient and valued auxiliary when two or more are in attendance from the same household. One takes the chair and the other is put in charge of "Tom, the elevator man," as he is familiarly known; the prospect of a ride after a short sitting is a balm much superior to Naboli or Cocaine in obtunding properties. A sweet and ideal little girl patient invites the use of the dental engine, ingenuously remarking, "because I like to hear it buzz," this trifling favor is usually accorded. Thoughtful and philosophical minds are sometimes found among our diminutive patrons. A case in point is that of little Mary, (four years of age) daughter of a college professor. She needed no bribes, and when told that it was best to have her baby teeth filled to prevent aching and keep the space for the permanent ones, opened wide her little mouth and had five fillings inserted without a murmur.

Another, that of a tiny lady of six summers is typical.

She must ask at least two questions or relate a rare bit of family news at every change. Attempts at any other course have proved fruitless.

Some faithful mothers sit upon the foot-rest and by dint of lavish flattery seasoned with timely correction, keep the peace.

However, some of the class under consideration deport themselves better if unaccompanied. The fact of being alone and thrown on their own resources serves to stimulate and develop native pluck.

Not infrequently the mild threat of "telling father," in an obstreperous case, acts almost magically as a quieter.

Politic parents have an adjustable method to suit the varying characteristics of their children. To one, "you must;" to another, "if you please;" while a third is promised a desired plaything, contingent on good behavior. Two little ones get ten cents for every cavity filled to place in their toy banks, and while the operation goes on they are beguiled by counting their gains.

One fortunate boy receives five dollars for every tooth extracted. This practice does not seem to beget a mercenary spirit as he never shows a disposition to double his premium.

The world is governed by a system of rewards and punishments. Adults are prone to forget that they were once children "pleased with a rattle, or tickled with a straw," and that this period should be suitably recognized and humored.

Patrons have asserted that, in their young days, a dentist was the embodiment of Tantalus and the forced visits to his office the most disagreeable of early trials.

Of course, this is wholly wrong, and argues a most inconsiderate and unwise treatment on the part of the dentist employed.

It cannot be denied that a kind of child exists with whom neither flattery, bribes, diversions, reasoning nor punishment will avail and recourse must be had to the "tour de force." However, with ordinary tact, this sort is not so numerous.

Great pains should be taken to create a favorable impression, failing in this, subsequent efforts may result in "Love's Labor Lost." Prejudices are difficult to overcome when the reasoning faculties are fully developed, well-nigh impossible before.

An important factor of success is, not to overtask the sitting to the extreme limit of childish endurance. A study may be made, with advantage, of each one's strength and disposition, and the manner and time gauged accordingly.

Early and proper chair education, (though trying and vexatious at times), well repays *some* dentists in after years.

Though the ethical relation of patient and practitioner be an unwritten law, yet, in equity, the former is under obligation to exercise self-control, that the service rendered be not needlessly fatiguing to the latter. Who could not testify to nerve and soul-trying cases where there was lack of confidence and sympathy. The inexplicable and unseen, but felt opposition to every movement doubling the necessary draft upon the vital forces and seemingly communicating the stiff and unyielding quality to the gold itself.

This is no figment of the imagination, for we have to do, not with wood and stone, but sentiment and complicated life structure involving occult physical and psychical forces.

Professionally, we are not alone responsible for those under our immediate care, but also for the far-reaching effects of rehearsals of dental experiences among schoolmates and friends. A pleased child will fancifully portray the first tooth trial with the pride of a victor, if he be affrighted, the dentist is ever after the largest of bugbears.

Doubtless a morbid fear does keep a great number of children from receiving dental services, and results in pain, ill health, and disfigurement.

There are no data from which to make an estimate of that number proportionate to the whole, but it is reasonable to infer that only a small minority ever enter our offices. It is, however, encouraging to note that owing to the faithful teaching and earnest efforts of some of the ablest and most experienced dentists the number is rapidly increasing.

Not the least among the benefits derived from child dentistry is the formation of the habit (and likely to be lifelong) of keeping the mouth cleanly and wholesome. With regret I feel impelled to state that the present condition of children's teeth is deplorable and appears to be growing worse—an opinion not hastily formed, but with due deliberation after eighteen years' practice. If true, it is a fact calculated to make the judicious grieve and the wise to anxiously enquire, what shall the end be?

Too many (dreading annoyance) dislike to operate for any but adults. But why evade responsibility and shirk professional duty when the efficacy and promise of great good lies in skillful attention to the deciduous teeth. Heredity teaches this unmistakably, and high civilization makes it imperative, if we would not have succeeding peoples edentulous.

This is not a pessimistic view, but one the truth of which forces itself upon every progressive and well-informed practitioner. I have examined the mouths of a score of little Sioux and Rees without discovering a defect in the temporary teeth; an inspection of an equal number of white children rarely reveals a perfect set.

The name of the ascribed causes of caries is legion, and the theoretical preventives equally numerous. The actual lesion and remedial treatment may be briefly summarized. Primarily unsymmetrical development of the intellectual faculties at the expense of physical vigor, resulting in imperfect innervation and defective nutrition. Hence, the teeth are not formed normally strong and resistant and readily yield to erosive agents, whether natural under the conditions, chemical, or parasitical.

As these organs belong to the tegumentary system, they, in a sense, sit at the second table, and often suffer from privation of nutriment.

Considering that we daily see such faulty teeth, of good type, but marred in the casting, are we not amenable at the bar of neglected duty, if like a faithful sentinel on the land or a watchman on the sea, we do not sound the note of alarm?

Begin with the young, as should all moral and social reforms, and by the aid of operative and prophylactic measures ward off threatened dangers.

Exorcise the evil spirits of doubt and fear which haunt too many offices and invite the gentle fairies which are near of kin to the German Zauber, who so charmingly rewards the good deeds of all his little friends.

Simple and narrow though the field is, with no romance in it anywhere, yet the end striven for ennobles it. After the spirit of Herbert's couplet:

"Who sweeps a room as for thy laws,
Makes that and the action fine."

By thus destroying the "little foxes," perfecting the technique and urging a hygienic mode of living, our profession may more effectually benefit mankind.

